# Let’s talk about work: pilot study of an education program on discussing work participation with cancer patients for general practitioners in training

**DOI:** 10.1186/s12909-024-05705-7

**Published:** 2024-07-09

**Authors:** Mariska de Wit, Kristel M. van Asselt, Marianne Mak-van der Vossen, Angela G.E.M. de Boer

**Affiliations:** 1 Public and Occupational Health, Amsterdam UMC location University of Amsterdam, Coronel Institute of Occupational Health, Meibergdreef 9, PO Box 22700, Amsterdam, 1100 DE The Netherlands; 2 Amsterdam Public Health research institute, Amsterdam, The Netherlands; 3 Department of General Practice and Nursing science, Julius Center for Health Sciences and Primary Care, University Medical Center Utrecht, Utrecht, The Netherlands; 4 Department of General Practice, Amsterdam UMC, Amsterdam, The Netherlands

**Keywords:** Education, Consultations, General practitioners, Cancer, Work

## Abstract

**Background:**

Cancer patients report that they lack support from healthcare providers when it comes to returning to or maintaining employment. In the education of general practitioners (GPs) in the Netherlands, there is little attention given to discussing work participation with patients. The aim of this pilot study was to evaluate a newly developed education program for GPs in training that focuses on discussing work participation with cancer patients.

**Methods:**

Two groups of in total twenty-one GPs in training participated in the education program. GPs were educated about the importance of discussing work participation with patients, work-related problems cancer patients can experience, and advice they can give to support cancer patients regarding work issues. In this pilot study using a mixed-method design, participants evaluated the program in two self-developed questionnaires and in a focus group discussion.

**Results:**

Seventeen participating GPs (81%) indicated that the education program was suitable for implementation in the education curriculum. Eleven participants (52%) reported that they had never discussed work participation with cancer patients before. Directly after the education program, eighteen participants (86%) reported that they planned to discuss work participation more often with their patients. Four months after the program, 67% indicated they had applied their new knowledge and skills in practice by discussing work participation and by referring cancer patients to occupational health professionals or online resources. According to the GPs in training, integrating the topic of work participation into other education for GPs in training and focusing on a broader group of patients could improve the impact of the education program.

**Conclusions:**

According to the results of this pilot study, the newly developed education program increased the awareness of GPs in training on the importance of discussing work participation with cancer patients. Future studies should focus on whether cancer patients experience more support from their GPs for maintaining and returning to employment after their GP has participated in the training program.

**Supplementary Information:**

The online version contains supplementary material available at 10.1186/s12909-024-05705-7.

## Background

Due to better cancer screening, earlier detection, and improved cancer treatments, cancer mortality has decreased in the past 50 years [1]. Cancer patients live longer, but still have to deal with the long-term consequences of their disease and accompanying treatments. When cancer patients return to work, they can experience physical and psychosocial problems that can negatively impact their work participation [2–4]. Examples of these problems are cognitive limitations, fatigue, increased susceptibility to infections, depression, and pain. It is therefore not surprising that cancer patients often work fewer days, take more sick leave, and have a higher chance of leaving paid employment compared to healthy workers [2, 5, 6]. Work is very important for people, because it provides financial independence, connection to others, and a better overall quality of life [4, 7]. Additionally, work can provide patients with distraction from their cancer treatment and help them to feel normal again [4].

In the Netherlands, occupational physicians have an important role in helping employees maintain employment or return to work after sick leave [8, 9]. However, many cancer patients do not have contact with an occupational physician [10, 11]. A previous study among cancer patients showed that only 30% did have contact with an occupational physician [11]. Furthermore, cancer patients in the Netherlands may not have access to an occupational physician if they are, for example currently unemployed or self-employed.

If patients do not have contact with an occupational physician, general practitioners (GPs) may be able to support them to increase their work participation [12]. In the Netherlands, GPs do not have a formal role in guiding cancer patients because the diagnostic, treatment, and rehabilitation phases take place in a hospital. However, the Dutch College of General Practitioners recommends that GPs take an active role in the psychosocial support for cancer patients. Nevertheless, cancer patients often report that they lack psychosocial support and support around returning to or maintaining employment. Results of previous studies show that cancer patients expect their GPs to support them in counselling and referral to other healthcare professionals that can help them to return to work [12–14]. Occupational physicians have also indicated that they expect GPs to take a proactive role when it comes to work guidance and referring cancer patients to occupational physicians [15]. Despite this, cancer patients experience very limited or no guidance from GPs [13]. GPs themselves have reported that they do not always discuss work participation with patients [15]. A possible reason for this is that the availability of guidelines for GPs on work participation is limited, and they therefore do not perceive it as their task to discuss work participation [12, 13]. Moreover, GPs often feel that they lack expertise and do not have sufficient knowledge about work regulations and legislation to advice patients on work issues [12, 15–17].

For GPs to support cancer patients with their work participation, it is important that they become more aware of the importance of discussing work participation and also be equipped with sufficient knowledge and expertise to advice these patients. GPs need to be aware of the types of problems cancer patients can experience during work participation and what kind of advice they can offer them for overcoming these problems. However, until now very little attention has been given to discussing work participation in the education of GPs in the Netherlands. The aim of this pilot study was to evaluate a newly developed education program for GPs in training that is focused on discussing work participation with cancer patients.

## Methods

### Research design

This study was a pilot study to evaluate the education program on discussing work participation using a mixed methods design by collecting both quantitative and qualitative data. Quantitative data was collected by inviting participants to complete two questionnaires to evaluate the education program. This was supplemented by a follow-up focus group representing the qualitative data to obtain more in-depth information from participants about their experiences and enhance understanding of the research findings [18, 19]. This study followed a constructivist paradigm, in which the outcome was a result of interaction between researchers and participants [20]. The Medical Ethics Review Committee of the Academic Medical Center confirmed that the Medical Research Involving Human Subjects Act (WMO) did not apply to this study and that an official approval by this committee was, therefore, not required (W22_255 # 22.311).

### Participants

Participants were GPs in training at the Amsterdam UMC who participated in the newly designed education program as a part of their education on cancer. The participants were in the third year of their education to become GPs. All participants signed an informed consent form for completing the questionnaires and for participating in the focus group discussion.

In the Netherlands, students must complete both a bachelor and a master’s program in medicine—a total of six years of education—to become a medical doctor. To become a GP, doctors must additionally complete a GP residency training of three years. In the first and third year of this educational program, the GPs in training work four days a week in a general practice where they deliver outpatient care (supervised by a senior GP). This is combined with one day a week of formal training activities in a university setting, designed to facilitate and deepen learning from the trainees’ experiences in practice. Residents are educated in small groups (of 10–15) about case histories, protocols, and skills, with dedicated time for collaborative reflection. In the second year, the GPs in training have internships in hospitals, nursing homes, and psychiatric clinics. As a result, all participants who are in their third year of the GP training have experience working in both general practice and inpatient facilities.

### Education program

The education program about discussing work participation with cancer patients was developed by the researchers (MdW, AdB, KvA) with the assistance of a GP who works as a teacher for GPs in training (SK). All the developers of the education program have experience providing education to physicians (in training). The content of the education program was based on different scientific studies and guidelines for physicians, such as the guideline for people with a chronic disease and work [2, 21–23]. Additionally, we used information from reliable websites with information for patients and physicians, such as the website of the Dutch Federation of Cancer Patients Organizations and the website of the Dutch College of General Practitioners [24, 25]. The objectives of the education program were formulated based on Bloom’s Taxonomy and are presented in Table 1 [26]. We applied the principles of constructive alignment to match the objectives with the correct learning activities, although there were no assessment tasks [27].

During the 90-minute education program, participants were educated about the importance of discussing work participation with patients. During the education program a PowerPoint presentation was used by the teacher. The first part of the program discussed why work is important for patients with a chronic disease in general (e.g., providing income, having social contacts, providing structure). Additionally, information was provided on the number of cancer patients that is not able to work full-time because of cancer and cancer treatments. It was also discussed that some people may not have access to an occupational physician to support them in returning to or maintaining employment, and that it is therefore even more important that a GP discusses work participation. In the second part of the training, participants were educated about the physical and psychosocial problems that cancer patients may experience that can impact their ability to work (e.g., fatigue, pain, concentration problems). In part three, the participants were taught what advice they could give to support patients seeking to return to or maintain work and which occupational health professionals could offer additional support to patients. In the fourth part of the training, participants were informed about a website for cancer patients who have questions about returning to or maintaining work [25], and in the fifth and final part of the education program, participants practiced discussing work participation during a simulated consultation. These simulated consultations were carried out in groups of three participants, with one playing the role of a GP, one playing the role of a patient with cancer, and one observing and providing feedback on the consultation. The case in the simulated consultation was described by one of the researchers (MdW) with input from two GPs (SK, KvA).

During the education program, participants were encouraged to interact. They exchanged views about the importance of work participation in their own lives and in the lives of workers with chronic diseases, their experiences discussing work participation during consultations, their experiences with cancer patients and their work participation, and their perceptions of the role of GPs after cancer treatment.

In the four months after participants participated in the education program, they received two emails to remind them to implement their new knowledge and skills in their practice.

**Table 1:** Content and objectives of the education program

Content	Objectives
Part 1: Importance of discussing work participation	1.1 Participants know the importance of work participation for patients with a chronic disease 1.2 Participants know the importance of discussing work participation with patients with a chronic disease
Part 2: Work-related problems for cancer patients	2.1 Participants know the physical problems that cancer patients can experience during work 2.2 Participants know the psychosocial problems that cancer patients can experience during work 2.3 Participants are aware of the fact that work can also have a negative influence on cancer patients
Part 3: Supporting cancer patients’ work participation	3.1 Participants know the roles of different health professionals who can support work participation for patients with chronic diseases 3.2 Participants are able to advise cancer patients about work participation 3.3 Participants know which professionals they can refer cancer patients to for supporting work participation and increasing fitness
Part 4: Website for cancer and work	4.1 Participants know about the website that can support cancer patients during work [25] 4.2 Participants know that they can refer cancer patients to the website for support
Part 5: Practicing a consultation	5.1 Participants are able to discuss work participation during a (simulated) consultation

### Procedure

Twenty-one GPs in training, divided over two separate groups, participated in the education program in September 2022. One week before the education program started, the participants received an email with information about the study to evaluate the education program. The education program was provided by one of the researchers (MdW), who has experience educating physicians (in training) and has a University Teaching Qualification.

#### Questionnaires

Directly after participating in the education program, participants signed an informed consent to participate in this study and completed the first questionnaire on paper. This first self-developed questionnaire, which was completed directly after participating in the education program, contained 35 questions. First, participants rated their overall satisfaction with the education program on a scale ranging from 1 (very dissatisfied) to 10 (very satisfied). In two open questions, they could state positive and negative elements of the education program, respectively. They also needed to report whether they thought the education program was suitable for the curriculum of GPs in training. The participants rated different statements about the importance of the education program, the program’s success in meeting its main objectives, and the program’s overall design. Finally, the questionnaire raised different questions concerning how participants would implement what they learned from the program in practice.

Four months after participating in the education program, they received an invitation by email to complete the second questionnaire online. The second self-developed questionnaire contained 22 questions. The questions asked whether and how participants had implemented the knowledge and skills they learned in the program in practice and, if not, why. The participants also rated different statements about the importance of the education program for practice.

#### Focus group discussion

The questionnaires were complemented with a semi-structured focus group discussion with participants to obtain more in-depth information about their experiences [18]. We chose to use a focus group discussion as an efficient way to collect different experiences and opinions regarding the feasibility of applying knowledge and skills in practice. All participants that participated in the education program and completed the first questionnaire were invited to participate in the focus groups by email. The focus group discussion was conducted with the participants approximately one week after participants completed the second questionnaire. The focus group discussion lasted one hour and was led by one of the researchers (MdW) with the assistance of a GP (SK). The focus group discussion was audio recorded. The participants who completed the two questionnaires and participated in the focus group discussion received a gift card of 20 euros as a thank you for participation.

### Analysis

Results of the questionnaire study were analyzed using descriptive analysis in SPSS [28]. Frequencies, percentages, means and medians were used to describe the data. The audio record of the focus group discussion was transcribed verbatim by one of the researchers (MdW) and then MAXQDA 2022 was used to assign codes to segments of the transcript using qualitative content analysis [29, 30]. For assigning codes to the transcripts, we used a combination of inductive coding and deductive coding based on the questions we asked during the focus group discussion. A first draft of the coding framework was developed by the same researcher (MdW) to describe the main themes and subthemes of the focus group discussion. Two other researchers (AdB, KvA) independently checked the codes and framework and added or changed codes or themes when necessary. The codes and coding framework were discussed among the three researchers (MdW, AdB, KvA) until consensus about the final coding of the transcripts and the coding framework was reached.

## Results

### Participants

In total twenty-one GPs in training at the Amsterdam UMC participated in the education program. All participants were in their third year of the GP training. Among the participants were five men and sixteen women. All participants completed the first questionnaire. Twelve participants (response rate 57%), among which eight were women and four were men, completed the second questionnaire four months after participating in the education program. Six of these participants (four women and two men) also participated in the focus group discussion one week after completing the second questionnaire. The other participants were not available to participate or did not want to participate in the focus group discussion.

### Questionnaire directly after participating in the education program

The twenty-one participants who completed the first questionnaire (response rate 100%) rated their overall satisfaction with the education program with a mean score of 6.9 and a median score of 7 on a scale from 1 (very dissatisfied) to 10 (very satisfied). The participants indicated that most learning objectives of the education program were met (Table 2). Nineteen participants (90%) indicated that they agreed or strongly agreed that they would be able to use the new knowledge and skills in their practice. These nineteen participants (90%) also agreed or strongly agreed that what they had learned would be useful for consultations with cancer patients and patients with other chronic diseases. Seventeen of the twenty-one participants (81%) thought that the education program was suitable for implementation in the curriculum of education for GPs. Eleven participants (52%) indicated that they had never discussed work with cancer patients prior and five participants (24%) also indicated that they did not discuss work with patients with other chronic diseases. Eighteen participants (86%) said that they were planning to discuss work more often with patients after participating in the education program. Fifteen participants (71%) were planning to advise patients more often about work. Almost all participants (95%) indicated that they were planning to advise cancer patients more often about other occupational health professionals who could help them to improve work participation.

**Table 2:** Scores on statements concerning the learning objectives (*N* = 21)

To what extent do you agree with the following statements about the learning objectives of the education program?	Totally disagree	Disagree	Neither agree nor disagree	Agree	Strongly agree
1. I know why work is important for people with a chronic disease.				14 (67%)	7 (33%)
2. I know the importance of discussing work with people with a chronic disease.			1 (5%)	15 (71%)	5 (24%)
3. I know what problems cancer patients can experience at work.		1 (5%)	1 (5%)	17 (81%)	2 (10%)
4. I know the role of different professionals in the field of cancer patients and work.			5 (24%)	13 (62%)	3 (14%)
5. I am able to advise cancer patients regarding work.		1 (5%)	2 (10%)	15 (71%)	3 (14%)
6. I know which professionals I could refer cancer patients to.			1 (5%)	15 (71%)	5 (24%)
7. I know about the website that can support cancer patients during work.			1 (5%)	9 (43%)	11 (52%)

### Questionnaire four months after participating in the education program

Twelve participants completed the second questionnaire four months after participating in the education program. Eight of the participants who completed the second questionnaire (67%) indicated that they had applied the new knowledge and skills they learned in the training in practice. The four participants (33%) who indicated in the questionnaire that they did not apply the knowledge and skills, said they had not seen patients in their practice for which they could use the knowledge and skills. However, all four of these participants stated that they would use the knowledge and skills if they did see cancer patients during consultations.

Seven of the twelve participants (58%) agreed or totally agreed that the knowledge and skills they had learned were useful for consultations with patients with other chronic diseases. Six (50%) had discussed work participation with cancer patients and seven (58%) had discussed work participation with patients with other chronic diseases. Four participants (33%) agreed with the statement that they discussed work participation more often with patients than before participating in the education program.

Three of the twelve participants (25%) agreed with the statement that they advised workers more often about maintaining and returning employment than before participating. Eight of the twelve participants (67%) reported giving patients advice about other professionals that could offer support, and three of them (25%) agreed with the statement that they referred patients to other occupational health professionals more often than before participating in the education program.

### Focus group discussion

After completing the second questionnaire, six participants also participated in the focus group discussion on the impact of the education program, factors that promote and limit discussing work participation with patients, and methods for increasing the impact of the education program. All main themes, subthemes, and corresponding quotes from the focus group discussion are presented in Additional File 1.

#### Impact of the education program

According to the participants in the focus group discussions, the education program reminded them to discuss work participation and made them more aware of the importance of work for patients.

*Quote P3: “What I did find an eye-opener was when we talked in the beginning about why people find work so important, you know. What it provides for people or… I thought it was good to be consciously aware of all those people who come into your office who… Yes, I’m also at work for the majority of my day. So, it’s just a really significant part of people’s lives.”*

The participants indicated that the education program also made them more aware of the problems that patients can experience that may impact work participation. Some of the participants said that because of the education program they discussed work participation more often with patients:

*Quote P4: “I do feel like since that education, I inquire more about work in case of these kinds of complaints.”*

Participants mentioned that the education program made them more aware about the possibility of referring patients to occupational physicians. In addition, the education program made them aware of the existence of an occupational physician that is specialized in cancer, which none of the participants in the focus group discussion had known about prior. Two participants in the focus group discussion mentioned that they referred patients to the website with information for cancer patients about work participation more often than they did before.

#### Promoting and limiting factors for discussing work with patients

Factors that participants reported to increase the likelihood of talking to patients about work participation included if there was a clear link between work and the health complaints, if they (the GPs) wanted to get a better picture of the patient, or if they had the idea that discussing work participation would help the patient. If discussing work participation would get more attention in guidelines or from the Dutch College for General Practitioners, for example, the topic would be seen as more of a standard, which would make it more likely for the GPs to focus on it.

*Quote P5: “And then, you see, if there are no further consequences attached, you won’t do it that well, but if perhaps in the NHG (Dutch College of General Practitioners) more attention is given to why you’re asking that, then you’ll also start doing it more quickly.”*

Limiting factors for discussing work participation with patients included some participants believing that the topic was not relevant for every patient they saw, especially when the patient did not bring up work or when the GP in training felt that discussing work participation would not help the patient. Some participants simply did not consider discussing work participation to be a priority, especially when consultations are short. Others mentioned that they just forgot to discuss work because it was not yet a routine question. Still others thought that discussing work participation was not the task of the GP but the task of, for example, the employer. They thought that they were not allowed to advise about work as a GP. Some participants in the focus group discussion who hadn’t provided advice about maintaining or returning to employment mentioned that they were insecure about what to advise regarding work participation.

*Quote P5: “I also notice in myself that there’s a barrier of: oh yeah, what am I supposed to do with all that information, and I can’t solve it at all. And I don’t know enough about it.”*

#### How to improve the impact of the education program

Although the education program encouraged participants to discuss work participation more often during consultations, some adaptations could improve the impact of the program according to the participants. They indicated that it is important to make clear in the education program why discussing work participation is important for the patient, as well as to discuss different cases, and to include information about laws and regulations when it comes to returning to work.

*Quote P4: “When you have a meeting about laws and regulations and what the patient needs, what their rights and obligations are. That also concerns us at the same time because we are also employees. We find it quite interesting, I think, to know more about that.”*

Likewise, incorporating the topic of work participation into other education that GPs in training receive and focusing it on a broader group of patients could, according to participants, help to increase the awareness of the need to discuss work. Other participants recommended offering the education program as an elective course and involving other occupational health professionals in delivering the education, so they can educate GPs in training about their professional expertise.

## Discussion

In this pilot study we evaluated a newly developed education program for GPs in training focused on discussing work participation with cancer patients. Directly after participating in the education program, 86% of the participants said that they were planning to discuss work participation more often with patients. Four months after participating, the majority of participants thought that their newly learned knowledge and skills were useful for consultations. Six of the twelve participants had discussed work with cancer patients. Some had also advised patients about maintaining or returning to employment, about other occupational health professionals, or about the website with information for patients. Still, some participants did not regard discussing work participation as their task or their priority during consultations. Some of them were insecure about what to advise regarding work participation. According to the GPs in training, integrating the topic work participation into other education of the participants and focusing it on a broader group of patients could improve the impact of the education program.

GPs have an important role in providing psychosocial care for cancer patients [31, 32]. However, psychosocial functioning is frequently understood as relationships, mood, and fatigue, whereas work issues are often neglected. In our study, some GPs mentioned that even after participating in the education program, they did not feel that discussing work participation is their task during consultations. Particularly when consultation times are short, discussing work participation does not take priority for them. Although participants obtained more knowledge and skills to discuss work participation with cancer patients, not every GP’s attitude towards discussing work participation changed. A change in attitude, in which someone acknowledges a need for the use of knowledge and skills, is necessary for behavior change [33]. However, changing a person’s attitude takes time and effort, and this change may not have been possible to accomplish with just one education program. The attitude that GPs in training have towards discussing work participation as part of psychosocial functioning is therefore something that needs more attention in order to change their behavior. More focus on employment among cancer patients from the Dutch College of General Practitioners, throughout the education of GPs, and within guidelines for these physicians might be helpful for this.

The GPs also mentioned that when patients do not initiate conversations about work participation, GPs tend not to give advice regarding the topic. These results were supported by a previous study by De Kock et al. [34] which indicated that GPs do not consistently ask about work during consultations. Since there is no structured care-path in primary care for patients after cancer treatment, GPs work is mainly demand-driven [35]. Whether or not they keep in touch during and after the treatment trajectory depends on the interests of patients and GPs. GPs expect cancer patients to talk and give a clear presentation of their complaints and let the GP know their needs during care [35]. However, some patients might not be aware that they can ask GPs about problems they experience when returning to work or during work or might not be aware that they could ask for a referral to other healthcare professionals. When patients are not aware that they can ask their GP about work and the GP does not actively ask about work himself, the topic will be ignored. It might therefore be helpful to not only raise GPs’ awareness of the importance of discussing work participation but to also inform cancer patients that they can discuss work participation with their GPs.

### Strengths and limitations

A strength of this study is that researchers with experience providing education to physicians and an experienced GP were involved in the development of the education program. Another strength of this study is that we conducted evaluations immediately and again four months after the GPs participated in the program. In this way, we obtained insight into how participants experienced the training program itself and how useful it was for them in practice. Another positive aspect is that in addition to questionnaires, we also conducted a focus group discussion to gain a more in-depth insight into the experiences of the GPs when implementing the knowledge and skills in practice.

However, there were also some limitations of this study. First, the number of participants who completed the follow-up questionnaire and who participated in the focus group discussions was limited. This limits the generalizability of the study results. Second, the questionnaires we used to evaluate the education program were self-developed and no validated questionnaires. Another limitation is that there were participants who did not see cancer patients during their consultations in the four months following their participation in the education program. Therefore, they did not have the possibility to apply their newly acquired knowledge and skills. Sending the follow-up questionnaire after a longer period of time could have prevented this. Finally, this study was a self-report study in which the GPs in training had to indicate the extent to which they applied the knowledge and skills in practice themselves. Observation of the GPs in training during consultations could be a more valid method to study this.

### Implications for future practice and research

The results of this pilot study suggest that the education program could be implemented in the curriculum of education for GPs in the Netherlands to enable GPs to better support cancer patients with their work participation. The content of the training program could be improved by making clearer why discussing work participation is important for GPs, discussing different cases, and including information about laws and regulations regarding return to work. Focusing on a broader group of patients, could also improve the impact of the training program. Moreover, integrating the topic of work participation into other parts of GP education and incorporating more focus on discussing work participation in guidelines for GPs might help to change the GPs’ attitudes towards discussing work participation. Additionally, it is important to increase awareness among cancer patients that they can seek advice from their GP regarding maintaining employment or returning to work. This is particularly crucial because the care provided by GPs to cancer patients is demand-driven. Therefore, a poster or an information leaflet with this information in the doctor’s office, for example, could prove helpful.

For future studies, we would recommend to study the effects of the education program on discussing work participation with cancer patients in a randomized controlled trial. It would be interesting to observe GPs during consultations or to study whether cancer patients experience a greater emphasis on work participation during consultations after their GP participates in the training. In this study we namely only asked GPs in training about their experiences with using the learned knowledge and skills in practice. Future studies should also be conducted to see whether the GPs are effective in helping the cancer patients to maintain or return to work after sick leave.

## Conclusions

The newly developed education program increased the awareness of GPs in training on the importance of discussing work participation with cancer patients. The majority of participants thought the knowledge and skills were useful for consultations with cancer patients and patients with other chronic diseases. Overall, the GPs thought that the education program was suitable for implementation in the curriculum of education for GPs and that attention to work participation should be repeated more often during the GP residency training. Four months after the training, the majority of the GPs had used the knowledge and skills they acquired during the program in practice. Half of the participants had discussed work participation with cancer patients. Because we had a limited number of participants and used self-report questionnaires in this study, future studies should focus on evaluating this education program on a larger scale and should observe GPs in training in practice to study the impact of the education program. Future studies should also focus on whether patients experience more support from their GPs for maintaining and returning to work after their GP participated in the training program.

### Electronic supplementary material

Below is the link to the electronic supplementary material.

Supplementary Material 1

## Data Availability

The anonymized data used and/or analyzed during the current study are available from the corresponding author on reasonable request.

## Abbreviations

GP: General practitioner
WMO: Medical Research Involving Human Subjects Act
