# Supplementary material for: Let’s talk about work: pilot study of an education program on discussing work participation with cancer patients for general practitioners in training
Source: BMC Med Educ. 2024 Jul 9;24:739. doi: 10.1186/s12909-024-05705-7 (PMC11232123; doi:10.1186/s12909-024-05705-7)
Supplement: Supplementary file 1 — Supplementary Material 1 [file 12909_2024_5705_MOESM1_ESM.docx]

**ADDITIONAL FILE 1.**

Table 1. Impact of the education program

| **Main theme** | **Subtheme** | **Quote** |
| --- | --- | --- |
| Impact of the education program | Reminds you to discuss work participation with patients more often. | P4: "I do recognize myself a lot in what she said, that indeed it comes a bit more to your frontal cortex. It's more in the front of your mind. So, I did find myself thinking about it more often." |
|  | Makes you aware of the importance of discussing work participation with patients | P1: "But I did find it to be a good reminder that it's important to discuss." |
|  | Makes you aware of the existence of occupational physicians specialized in cancer (BACO) | Interviewer: "That (occupational physician that is specialized in cancer) was really new for you, right?"  P6: "Yes, I'd never heard of it before." |
|  | Makes you aware of the importance of work participation for patients | P3: "What I did find an eye-opener was when we talked in the beginning about why people find work so important, you know. What it provides for people or... I thought it was good to be consciously aware of all those people who come into your office who... Yes, I'm also at work for the majority of my day. So, it's just a really significant part of people's lives." |
|  | Makes you aware of the possibility to refer a patient to an occupational physician | P1: "But I think the education does make us more aware that we can refer people to an occupational physician as well." |
|  | Does not increase the number of times GPs discuss work participation with patients | P3: "But it hasn't really prompted me to ask about it even more now. No, not that." |
|  | Increases the number of times GPs discuss work participation with patients | P4: "I do feel like since that education, I inquire more about work in case of these kinds of complaints." |
|  | Increases the number of times GPs refer patients to the website about cancer and work | P1: "And then you do refer to the website with information about cancer and work or to an occupational physician. It did help me with that." |
|  | Makes you aware of the problems that patients can experience that may impact work participation | P2: "I think it has positively contributed to the reminder and understanding of what people can struggle with alongside the physical issues. All the things that come into play." |

Table 2. Promoting and limiting factors for discussing work participation with patients

| **Main theme** | **Subtheme** | **Quote** |
| --- | --- | --- |
| Promoting factors for discussing work participation with patients | If you want to get a better picture of the patient | P5: "You often ask, especially for the sake of your own impression: what do you do for a living? What sports do you engage in? For example. You do ask that quite often." |
|  | If your goal is to monitor or guide the patient | P4: "And we keep in touch with that patient every week, every two weeks, every three weeks, every month – we keep calling, monitoring proactively. Like, hey, how are you doing? And through this, much more comes out. For instance, well, I did end up taking sick leave from work because the team no longer understands. And then you have starting points." |
|  | If you have the idea that discussing work participation would help the patient | P5: "See, I can ask every hypertension patient how their work is going and probably 9 out of 10 will respond: yeah, it's fine, no problem. Then you stop asking. So, it should yield something as well. Because otherwise, you're back to it, you lose it again, and you think: yeah, it's nice, but why should I ask it every time." |
|  | If you have your practice with more (new) patients | P2: "As a physician in training, of course, you might have fewer new cancer patients in a shorter time, fewer new (...) So if you perhaps have a regular practice, that also happens more naturally or something. And that comes from within yourself." |
|  | If discussing work participation becomes a habit/standard | P5: "Actually, you want it to become a habit so that you're aware of it, that it can achieve something, yield results. Then you start doing it a bit more." |
|  | If discussing work participation will also get attention in guidelines/ Dutch College of General Practitioners | P5: "And then, you see, if there are no further consequences attached, you won't do it that well, but if perhaps in the NHG (Dutch College of General Practitioners) more attention is given to why you're asking that, then you'll also start doing it more quickly." |
|  | If there is a clear link between work and the health complaints | P3: "But just more to have a picture of who is sitting in front of me." |
| Limiting factors for discussing work participation with patients | Not seeing many cancer patients | P5: "I don't think I've seen a lot of cancer patients in general." |
|  | Having the idea that there is a lot to be discussed and work does not have the priority | P4: "And work is a bit of a neglected aspect in that." |
|  | Having short consultations | P4: "It's quite challenging for us to truly understand what the patient's life looks like with this condition. It's simply difficult to gain a comprehensive picture from a single fifteen-minute consultation." |
|  | Having the idea that discussing work participation does not help the patient | P5: "And also because I wonder, is it relevant? Will it yield something that we can work with or that can help a patient? Because I think, especially with chronic illnesses, many people can function well. And then you'll often just get, yeah, no particular issues. And then it doesn't yield anything more for you, so you often let that go as well." |
|  | Being insecure about what you can advise regarding work participation | P5: "I also notice in myself that there's a barrier of: oh yeah, what am I supposed to do with all that information, and I can't solve it at all. And I don't know enough about it." |
|  | Forgetting to discuss work participation | P4: "but then it also fades away somewhere again. Like what happens with a lot of education, I think. You hear something very interesting about a topic, and then you're like oh, I'm really going to pay attention to that, and then it fades away a bit again." |
|  | Discussing work participation is not a standard/habit | P5: "Probably because you just don't do it often enough." |
|  | Having the idea that discussing work participation is not relevant for every patient you see | P4: "And yes, the people, I've had a hypertension patient before, and that relationship with work, I don't really see it." |
|  | Having the idea that initiating a discussion about work participation is the responsibility of the patient | P3: "Well, if you have a chronic illness, I think it's your own responsibility to discuss that at work and to look at it like: I have this now, what aspects of my life does this affect?" |
|  | Having the idea that work is not the main reason for the patient to consult the GP | P2: "It's definitely not unwillingness to not bring it (work) up, but initially you prioritize the patient because they have a question." |
|  | Having the idea that patients discuss work participation with their employer | P5: "Yes, and for me, a reason not to specifically ask: how is it going at work? Is because I also kind of have the expectation that it's something between the relationship of the employee and the employer and that it's between them. Yeah, I do have that expectation. That it will come out and be discussed between them at some point." |
|  | Having the idea that discussing work participation is not a task for GPs | P2: "So, I've always been inclined to say that as a general practitioner, it's not our place to say what you should do with work. So, you really need to have that conversation with someone else." |
|  | Having the idea that you cannot or are not allowed to advise about work participation as a GP | P2: "I'm not allowed to say anything about that (work), and I do say that, that's not my role. I'm not allowed to say what I would advise you on how to handle that at work. It also stems from that." |

Table 3. How to improve the impact of the education program

| **Main theme** | **Subtheme** | **Quote** |
| --- | --- | --- |
| How to improve the impact of the education program | Making clear why discussing work participation is important for general practitioners | P5: "Yes, especially also because of the consequences attached to it. Because often, when you ask those questions about work or something, the response is: just fine. So, it's nice that you asked, but what... so you want to know, like, how's it really going for a diabetic? You want a bit more, you know, the purpose, why are you asking it? What do I gain from asking this question?" |
|  | Adding discussions about different cases | P4: "Yes, and what would you say? What would you advise? I think that could really be interesting. And meaningful. Because work is often, well, the biggest part of our lives is spent working. So, it's really quite relevant." |
|  | Including information about laws and regulations | P4: "When you have a meeting about laws and regulations and what the patient needs, what their rights and obligations are. That also concerns us at the same time because we are also employees. We find it quite interesting, I think, to know more about that." |
|  | Focusing the education on a wider group of patients | P1: "But I think in terms of education, it was very, it was very focused on cancer and work. And I was wondering if it might just be better to provide education about discussing work with your patients in general." |
|  | Integrating the topic of the education program into other education | P5: "There just needs to be more awareness, but that can't come from a single educational moment. It should be integrated throughout the three years. This is also the case with diabetes or hypertension. (...) It might be beneficial to have it mentioned more consistently across all subjects, like hey, ask about their work too." |
|  | Offering the education program as an elective course | P1: "I would actually find it to be an nice elective course. Compared to some other elective courses, I think I find this quite interesting."  P5: "I think there would be enough people enrolling for it." |
|  | Including other occupational health professionals in providing the education | P4: "(...) and that you also maybe include some other experts to make it a bit more engaging." |
